# The invisible burden of managing COVID-19 for Australian women: Cognitive labor and public health information

**DOI:** 10.3389/fpubh.2023.1041944

**Published:** 2023-02-01

**Authors:** Ashlin Lee, Naomi Kakoschke, Liesel Higgins, Andrew Reeson, Emily Brindal

**Affiliations:** ^1^Environmental Informatics Group, Environment Department, Commonwealth Science and Industrial Research Organisation (CSIRO), Canberra, ACT, Australia; ^2^Justice and Technoscience Lab (JusTech), School of Regulation and Global Governance, The Australian National University, Canberra, ACT, Australia; ^3^Human Health, Health and Biosecurity Department, Commonwealth Science and Industrial Research Organisation (CSIRO), Adelaide, SA, Australia; ^4^Australian E-Health Research Centre, Health and Biosecurity Department, Commonwealth Science and Industrial Research Organisation (CSIRO), Brisbane, QLD, Australia; ^5^Humans and Machines Department, Data61, Commonwealth Science and Industrial Research Organisation (CSIRO), Canberra, ACT, Australia

**Keywords:** cognitive labor, information seeking, COVID-19, mental load, public health

## Abstract

Providing accurate and timely public health information is an ongoing challenge for public health officials. The COVID-19 pandemic has exacerbated such challenges and presented unique difficulties in providing public health information, through the parallel rise of an “infodemic” of mis/dis-information. Understanding why individuals select, use and change their public health information seeking behaviors around COVID-19, and the relationship of these decisions relative to participant characteristics, is therefore an important step in understanding and responding to infodemics. This study used a qualitative survey (*n* = 255) and free-text qualitative questions to ask (1) Why participants use an information source, (2) How participants used an information source, and (3) How information seeking behavior has changed since the COVID-19 pandemic. Participants were primarily women, born in Australia, with *de-facto*/married relationships, without children at home, with university/college qualifications, and employed full-time or unemployed/retired. Most participants identified “easiness” and “immediacy” as reasons why they chose and used information, with sources primarily used for planning, communication, and decision making. A minority of participants changed their information seeking behavior since the COVID-19 pandemic. Those who did change, desired more immediate and accurate information. Emergent themes of care and anxiety were also noted, raising questions around the impact of mental load and cognitive labor in some female populations. Women may be suffering from increased cognitive labor and a gendering of public health information seeking behavior in the context of COVID-19. The impact of these attributes on women requires greater empirical research and consideration amongst front line practitioners and public health professionals.

## Introduction

Providing accurate and timely public health information is an ongoing challenge for public health officials. The COVID-19 pandemic has exacerbated such challenges and presented unique difficulties in providing public health information. One such challenge is misinformation (i.e., misleading information), particularly through digital platforms, which has negatively influenced health behaviors such as vaccine uptake (1–3). As Stein et al. (4) note, the success of COVID-19 responses such as mask wearing and vaccination is contingent upon what Lippmann (5) calls the pseudo-environment, the mediatory space between an individual and their environment, where public opinion and other stimuli shape our perception of the world. Exposure to anti-vaccination messaging and other forms of health misinformation on these platforms has distorted the pseudo-environment for some populations. This has increased the presence of vaccine hesitant attitudes, that otherwise informed citizens have, toward vaccines (2), it has spurred conspiracy theories (6), and promoted false and dangerous treatments (7), all while degrading faith in proven measures like vaccines. As Knight et al. (8) note, whilst social media platforms can contribute to vaccine hesitancy, they also possess the potential to improve vaccine uptake. The latter suggests that the relationship between public health information sources and the public varies, and that information seeking behaviors might change depending on the context and medium (9). For example, users might be using different social media platforms to as a part of different information seeking behaviors; they may (hypothetically) be exposed to, and respond to, misinformation on Twitter, but seek out trustworthy sources on TikTok, depending on their external circumstances.

Relatedly, health information seeking during COVID-19 has been impacted by an over-abundance of information, or an “infodemic.” Excessive amounts of information make it more challenging to locate health-related information from trusted sources (9). A recent systematic review identified five key contributors to infodemics, including information sources, communication channels, and message content (3). However, there is currently little qualitative research regarding this.

Evidence suggests that information seeking sources may also vary depending on personal characteristics. For example, women have been shown to more frequently access public health related information than men, and to take the role of “health information gatekeepers” of their family (10). During the COVID-19 pandemic, the burden of care for women increased, whilst their wellbeing decreased (11). One potential reason for this is that women and mothers experienced a greater than usual “mental load” or “cognitive labor” particularly during lockdowns. These terms describe responsibilities such as planning, decision making, and monitoring, required as a part of everyday life (12, 13), for which women are disproportionately responsible even in the absence of a global pandemic (14). However, it remains unclear whether an increased burden of care is attributable to not only physical but cognitive dimensions of labor, and whether the latter could be attributed to increased health information seeking behavior.

The current study aimed to provide preliminary insights into why participants selected, used, and changed their sources of public health information during COVID-19, and the relationship of these decisions relative to participant characteristics.

## Methods

### Participants and design

Adults aged over 18 years who were currently residing in South Australia and not currently isolating or in lockdown were eligible to participate in the study. Participants were recruited *via* the CSIRO Nutrition and Health Clinic volunteer database (a standing database of interested research participants) to evaluate website content containing resources related to COVID-19 and then asked optional questions about information seeking. All volunteers in the stated database were invited to take part in the study. The current study employed a mixed-methods cross-sectional study design.

### Materials

This study employed an online survey comprising questions about public health information seeking behavior and COVID-19, which were included as part of a larger study evaluating a COVID-19 self-isolation preparedness checklist. Questions for this study subsection were developed through a brainstorm of potential COVID-19 information sources, which were distilled into clear categories and used as closed choice survey questions. These categories are consistent with other previous studies, and cover different online sources, social and mobile media sources, personal sources, and community sources (15–17). To ascertain the meaning behind these choices and collective qualitative data on these meanings, three open questions followed (see the “Open ended questions” section in the results). The other questions related to the COVID-19 preparedness materials are not relevant for this analysis.

### Procedure

The study was approved by the CSIRO Human Research Ethics Low Risk Review Panel (#2021_105_LR), before invitations were emailed to participants registered on an existing volunteer database. Interested participants accessed an anonymous online survey hosted on the Alchemer survey platform where consent and information sheets were provided. Participants then reviewed materials for the preparedness checklist (18) and completed a short feedback questionnaire relating to these materials. Information seeking questions were included at the end of the survey. Completing the survey took ~30 mins, and those who completed the survey were offered the chance to win 1 of 3 $100 AUD vouchers. The data was collected throughout December of 2021.

### Data analysis

The quantitative data are presented descriptively and compared using Chi-Square analyses. Chi-Square was applied to compare use (vs. not) across demographics including gender, age group, employment status, education level, marital status and whether participants were born in Australia (or not) for the top five information sources. Given repeated analyses, significance levels were adjusted to *p* < 0.01.

For the qualitative data, an abductive thematic analysis was conducted using NVivo 12 (Melbourne, Australia) with data constantly being reflected upon *vis-a-vis* existing theory and data. Results were generated using Green et al.'s (19) approach to achieving rigor in qualitative analysis. This consisted of (1) data immersion through reading and reflection, (2) open code generation, (3) sorting codes into descriptive categories, and (4) developing explanatory themes informed by appropriate theory.

## Results

### Participant characteristics

The survey was sent to ~18,000 participants on the volunteer database and a total of *N* = 438 commenced it (2.4%). Based on the eligibility screening questions, *n* = 61 participants were deemed ineligible from further participation due to not residing in South Australia (*n* = 15) or being in isolation or lockdown (*n* = 46). Of the remaining *n* = 377 participants, *n* = 123 had missing data. The final sample comprised *n* = 255 adults aged 18–84 years (female: 73.3%, *n* = 187). We received the majority of our results from women, born in Australia, with a *de facto* or married relationship status, without children living at home, with university/college qualifications, and employed full-time or unemployed. Sample characteristics are presented based on the sample included in the current research analysis (see Table 1).

**Table 1:** Participant characteristics for those who provided open-ended responses.

**Variables**	***n***	***%***	**Variables**	***n***	***%***
**Gender**			**Highest level of education**		
Male	65	25.5	Year 9 or below^b^	2	0.8
Female	187	73.3	Year 10^b^	16	6.3
Non-binary	2	0.8	Year 11^b^	13	5.1
Prefer not to answer	1	0.4	Year 12	20	7.8
**Born in Australia?**			Certificate 2 or 3^c^^*^	15	5.9
Yes	183	71.8	Certificate 4 or 5^c^^∧^	12	4.7
No	70	27.5	Diploma or advanced diploma	45	17.6
Prefer not to answer	2	0.8	Bachelor/ undergraduate degree	57	22.4
**Marital status**			Graduate diploma/graduate certificate^d^	20	7.8
Single	52	20.4	Postgraduate degree^d^	48	18.8
*De facto*/married	157	61.6	Certificate not further defined^c^^#^	5	2.0
Divorced^a^	37	14.5	Level not determined	2	0.8
Widowed^a^	9	3.5	**Employment status**		
**Child living with you?**			Part-time	42	16.5
Yes	75	29.4	Full-time	89	34.9
No	179	70.2	Casual	18	7.1
Prefer not to answer	1	0.4	Unemployed	104	40.8
			Prefer not to answer	2	0.8

a, b, c, d Shared superscripts indicate categories that were combined in further analyses.

* Basic or skilled vocational qualifications.

∧ An associate diploma or above.

# An undefined vocational qualification.

### COVID-19 information sources

**Figure 1:**
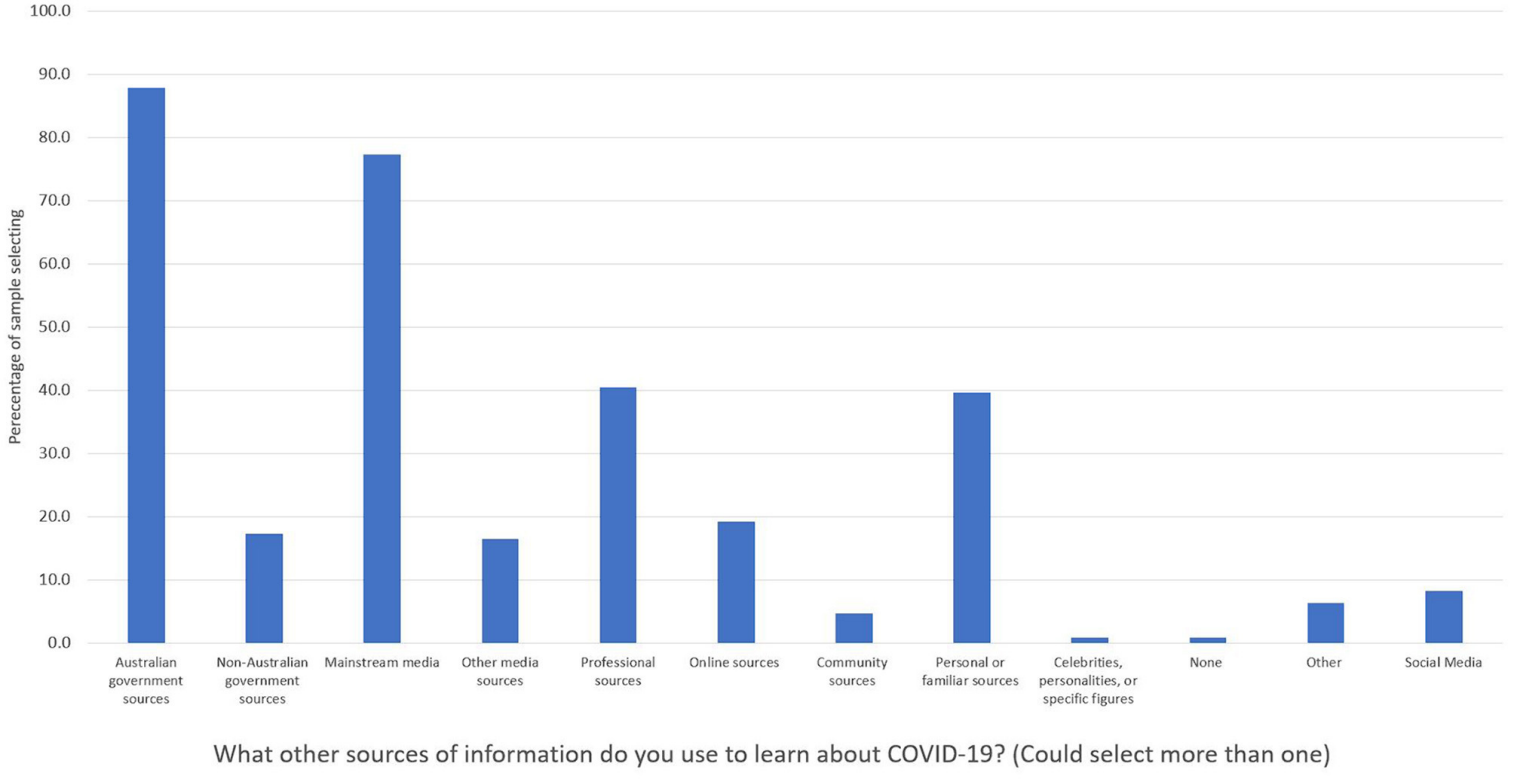
Percentage of people selecting (vs. not selecting) information sources used to learn about COVID-19. Participants (*n* = 255) could select multiple options.

Australian Government sources were the most widely used, with the majority of participants selecting this source type, followed by privately owned mainstream media (Figure 1). Professional sources, closely followed by personal/familiar sources were the next most used. “Other” responses included newspapers and workplaces.

For the top five information sources, use of Australian Government sources varied significantly by level of education (see Table 2 for inferential statistics). Participants who had not completed high school were less likely than expected to use Australian Government sources relative to the sample's average (64.5% vs. 87.8%). Use of mainstream media sources varied significantly by employment status. Participants with full-time employment were less likely than expected to use mainstream media sources relative to the sample average (62.9% vs. 77.3%).

**Table 2:** Chi-square values for comparisons between use (vs. not) of top five sources of information by key participant characteristics (*n* = 255).

**Characteristics**		**Sources of COVID-19 related information**									
		**Australian government**		**Mainstream media**		**Professional**		**Personal**		**Online**	
	**df**	**Chi-square**	***p***	**Chi-square**	***p***	**Chi-square**	***p***	**Chi-square**	***p***	**Chi-square**	***p***
Gender^a^	1, 252	0	>0.99	1.52	0.218	0.25	0.62	1.25	0.264	3.80	0.051
Age Group	9, 255	11.23	0.261	33.12	<.001	8.71	0.465	9.95	0.354	10.52	0.310
Marital status	2, 255	2.95	0.229	2.44	0.296	1.32	0.516	6.76	0.034	0.36	0.836
Employment status	3, 253	6.6	0.086	16.82	0.001	1.08	0.783	0.34	0.953	1.74	0.628
Level of education	5, 253	18.07	0.003	9.63	0.087	4.14	0.529	5.91	0.315	3.71	0.593
Australian born	1, 253	1.22	0.269	2.02	0.155	0.31	0.577	0.59	0.443	0.827	0.363

a Excludes non-binary due to small cell size (n = 3).

**Figure 2:**
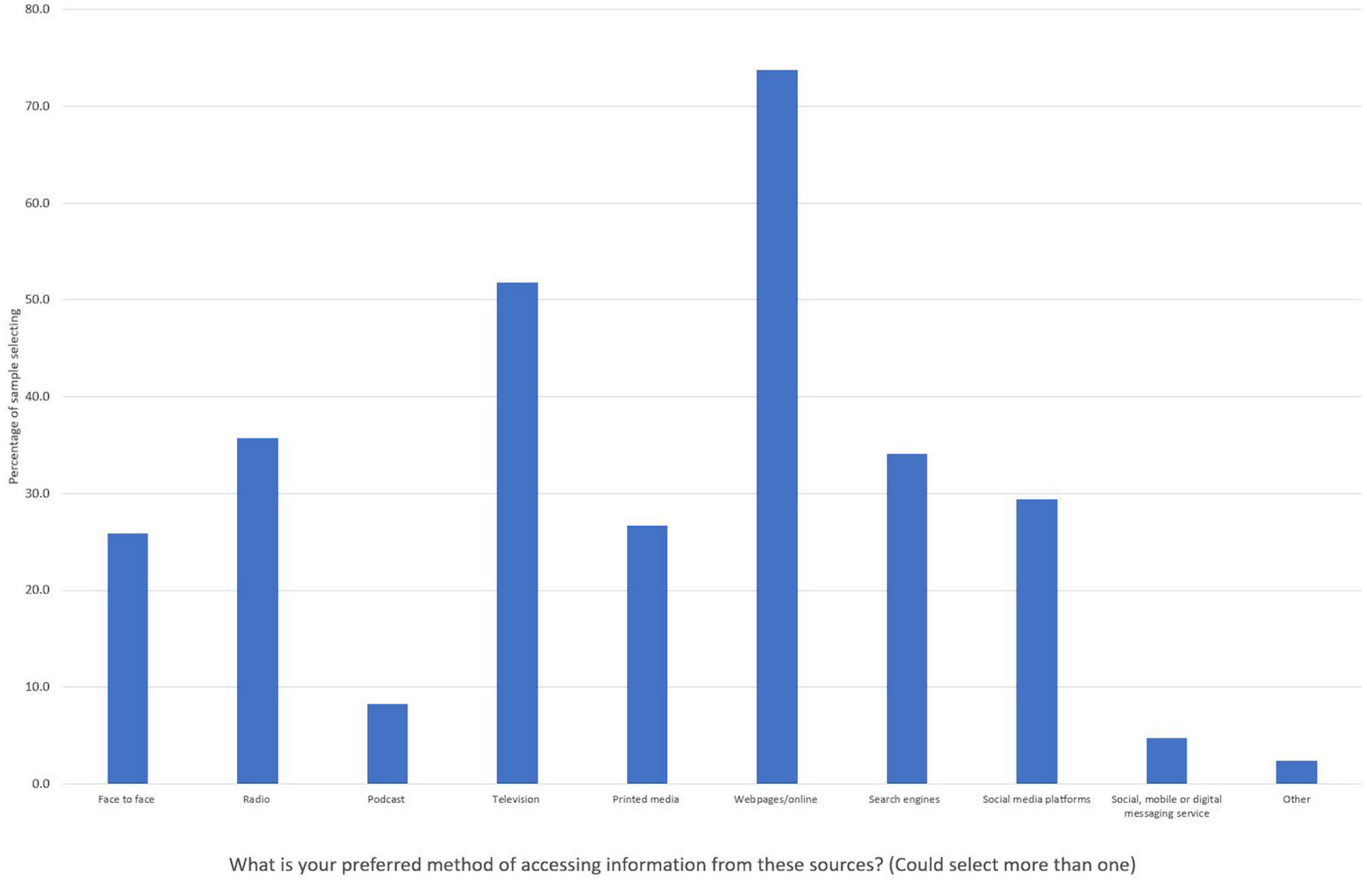
Percentage of method of access (vs. not) for different sources of information (*n* = 255).

Online resources were used by almost three quarters of the sample who preferred online methods for accessing information such as webpages, whilst only half of the sample used television (see Figure 2). Nevertheless, traditional media sources such as television, radio, and print are still relevant for much of the sample, and 38.0% (*n* = 95) used two to three of these sources in combination. Search engines were also amongst the key methods for accessing information about COVID-19.

### Open-ended questions

We observed a large gap in unique coding references for female participants compared to male participants, in line with the female bias of our sample. Comparison of coding frequency and gender illustrates this issue with Question 1 split 265 vs. 83, Question 2 split 179 to 61, and Question 3 split 364 vs. 124, all in favor of female vs. male participants.^1^ Thus, the qualitative results should be interpreted with caution as they may provide greater insights into female participants only.

#### Question 1: Why do you use these information sources?

Participants' responses focused on immediacy and quality. Immediacy (20) refers to whether information sources reflect convenience and easiness in their interaction with the participant. For example:

*Easier and can be accessed anywhere and anytime. The information is more current (Participant 34—F)*.

*Easily accessible. Simple, succinct to read. Links to other websites/news sources if I want to read more (Participant 16—F)*.

*They are available when I have the time. I can re-read things to better understand what is being said. I don't have to be polite to people (Participant 137—M)*.

Accessing and using preferred sources is not limited by time and space; they have desirable physical and user interface qualities (such as succinctness and privacy), and also easily connect to further content (i.e., links to other sources). These allow the preferred source to fit seamlessly into a participant's existing context.

The importance of connectivity amongst sources accentuates the theme of quality. Rapidly accessing information is a necessary condition for selecting a source, but there are also sufficient requirements reflecting the quality and validity of a source:

*The television news is easy to access and gives an overview of the current state of affairs. If I want to access more accurate information, I use the Government COVID app or website. I don't want to read “opinion” of people based on their fame. I only want facts and hope that the information I get from government websites reflect professional medical opinion (Participant 80—F)*.

*Easily accessible and substantially authoritative (Participant 225—M)*.

*Reliable, trusted, unbiased, every day, plus free sources (Participant 147—F)*.

*They are generally reliable with the best up to date information and can be cross checked (Participant 125—F)*.

There is no competition of themes here, but a contingency; sources are preferable only if they are truthful, but this truth needs to be easily accessible.

#### Question 2: How have you used these sources to respond to, or prepare for COVID-19 related events?

Remaining informed and coordinating responses to COVID-19 are the primary reasons that participants use the identified sources. Changing COVID-19 restrictions and outbreak locations meant participants leveraged accessible and trustworthy sources to stay abreast of a rapidly changing situation:

*I have checked government websites carefully to make sure I am following the latest COVID guidelines. People also share information socially but it's important to check the news and government websites to make sure the information is objective and accurate (Participant 228—F)*.

*Up to date and relevant information and keeping abreast of changes and how they will impact me and my family (Participant 89—F)*.

By staying up-to-date with changes, participants are able to better coordinate how they, or those immediately around them, are able to respond to the pandemic:

*Warnings for changes to restrictions, including mask requirements. Changing interstate travel plans. Locating vaccine suppliers and arranging appointments as well as being aware of rules changing for my employment (Participant 1—F)*.

*Kept up with outbreaks, local rules and stocked the house with essentials (Participant 114—F)*.

*Checked on restrictions when considering travelling interstate and what was required when arriving in another state. Also checking on where outbreaks may have occurred (Participant 145—F)*.

These coordination activities reveal the range of impacts individuals responded to during the pandemic. Rapidly changing, and sometimes complex, rules meant changes to individual and familial tasks. This includes the need to negotiate travel restrictions across different jurisdictions, a need to monitor potential exposure sites for infection risks, and changes to how to find appropriate medical care. Essential workers were especially vulnerable to these rapid changes.

#### Question 3: How has your information seeking behavior changed?

Like the way that accuracy is a contingent quality for choosing information sources, participants (*n* = 100) who indicated that they have changed their behavior indicated accuracy and truthfulness as a motivator:

*At the start of the pandemic, I read and listened to as much as possible about the situation. This included all media, Facebook posts, friends, and international news. These days I have concluded that there is too much misleading information and opinion-based information, so I try to look only to “official” sources. I hope that these sources are providing true information (Participant 80—F)*.

*Too much misinformation circulating on social media and in conversation. I seek out peer reviewed materials, and guidance from Health authorities (Participant 156—F)*.

Dual concerns of information overload and misinformation encouraged participants to change their practices, which often meant switching sources:

*Go straight to government sites. Do not use social platforms, e.g., Facebook (Participant 32—F)*.

Or changing the focus of their information seeking to topics of the most immediate saliency and importance:

*I seek to know less about the virus and how it's impacting other countries/states in terms of healthcare, welfare, social etc. I now just want to know local information like exposure sites etc. I just want quick facts now (Participant 16—F)*.

### Emergent themes

We also examined our data for any crosscutting, emerging themes. Although no all-encompassing narrative was found, we noted “care” and “anxiety” as themes worthy of further discussion in the context of COVID-19, especially given recent research on cognitive labor.

#### Care

The desire to care for others extended across multiple questions. For example, in responding to Question 2, participants described a consideration of those immediately around themselves, and the potential impacts of COVID-19 on these groups as a reason for their public health information seeking:

*Awareness of what might be brewing, instructions for workplace compliance, awareness of how my family might be feeling about issues (Participant 47—F)*.

*Consideration of risks to me and my family so we can try to be as safe as possible (Participant 142—F)*.

*[…]I am particularly mindful of my elderly mother whom I care for as if she were to contract the virus, is most likely to come from me given my work (Participant 229—F)*.

Female participants foregrounded care more often than males, with only one male participant mentioning family relationships (specifically to Question 3). While references to care understandably emphasize family, it does not exclude other situations, as Participant 47 notes compliance with their workplace's rules, as important in their information seeking rationales.

#### Anxiety

Participants also noted feeling overwhelmed and emotionally distressed at the amount and quality of information available to them, and consequently reduced their information seeking:

*In the beginning I was monitoring multiple sources of information as I wanted to know what was going on at all times. Over time I have reduced the number of sources I monitor, as I found it became overwhelming and I was spending too much time obsessing over every small detail (Participant 177—F)*.

*I try and limit any unofficial content because I don't trust the “noise.” I only read/watch what I have to because it makes me anxious to process too much (Participant 47—F)*.

The need to moderate the information one consumes also appears to merge with issues of care, as one participant succinctly described:

*I have kept myself and my family healthy, by not listening to the news I am not swept up into the stress and anxiety of everything, I try to understand how the virus works to the best of my knowledge through credible sources, kept my house stocked up and knowing we will have sufficient items during a lockdown (Participant 19—F)*.

Managing both the amount of information and emotional consequences of information overload is as important as keeping the participant and their family healthy, as selecting the right source. These choices play out through practical caregiving activities, such as managing the family's groceries.

## Discussion

Our study aimed to explore why individuals selected, used, and changed their public health information seeking behaviors in the context of COVID-19. The relationship of these decisions, relative to participant characteristics, was also explored. Responses were unintentionally skewed toward female, born in Australia, educated, older, married/de-facto participants, without children at home, and while not representative, do provide qualitative insights into how public health information is being experienced (21). This paper notes the following findings: (a) that source selection is motivated by easiness, immediacy and quality, (b) that people use sources to support decision making and to keep up-to-date, (c) that a minority of people have changed their information seeking behaviors and those who do, seek easiness or quality, and (d) that there are emergent themes of care and anxiety worthy of further investigation, especially around themes of cognitive labor.

Regarding information sources, the majority of people relied on online resources provided by the government or mainstream media. Our qualitative findings illustrate the continued importance of providing high quality public health information in an accessible and immediate way attuned to individuals' situational needs. The finding that only 100 participants (i.e., 39%) changed their information seeking behavior and are motived by the validity of information, aligns with existing research on public health information (22). We observed minimal gender differences in the sources and delivery of COVID-19 information, but did observe significant differences in level of education, with participants without a high school education less likely to use government information.

Crosstabulation of qualitative themes against demographic variables indicated that most information seeking practices and responses were provided by female participants across all three questions. While neither the emergent themes nor primary questions capture causal or representative answers, there are interesting questions raised concerning the intersections of gender, public health information seeking behaviors. Particularly regarding our understanding of the gendered nature of public health information behaviors in the context of COVID-19. By providing the greatest number of detailed responses, women seem more engaged in public health information seeking behaviors relating to COVID-19. However, this engagement may also connect to experiences of care provision and anxiety, which suggests women may (un)willingly assume a greater burden of responsibility for managing information during complex health situations such as the COVID-19 pandemic. These observations, however, should be considered in the context of limitations of this study (see below).

The latter findings connects to emerging research on concepts including cognitive labor (12) or mental load (13). In the context of family life, Daminger (12) notes that this labor can be gendered, with women taking on more work even when male partners are uniquely qualified in cognitive labor (for instance, when male partners were professional project managers). Dean et al. further develop this noting intersections with emotional labor (23), especially within the contexts of COVID-19 related lockdowns. Here, women were increasingly burdened with household chores, work-from-home employment, social isolation, and potentially childcare arrangements, in a way that was neither recognized or rewarded by decision makers or partners alike (24, 25).

In the context of this article and public health information behavior research more broadly, this finding raises questions around how cognitive labor mediates public health information. The need for accessible, quality information sources may reflect the greater burden women face in managing situational needs in the context of COVID-19 given that they are already mentally and emotionally overworked and may desire immediate and truthful answers. The minority result for changing information seeking behaviors may be viewed less as the preferred choice, but instead the reality that women lack the time and mental space to change their information seeking through reflection and deeper engagement. The latter may connect to the caring work that participants describe, and how additional burdens of care work are placing an increasing cognitive burden upon women in the context of COVID-19. Given that previous research (26) has established that women are more likely to respond to health information and seek appropriate medical care, establishing how an experience, like cognitive labor, impacts women is an important public health question, particularly given that public health relies heavily on health communication techniques. Those practicing in public health need to consider the invisible burden that excessive and constant change has, as well as how this could unequally impact those already being further burdened by the health issue.

The practical implications of these initial findings for public health professionals are that cognitive labor may mediate how public health information is being received by women, and that consideration should be given to whether this represents a gender disparity in public health. Given the resources that people often rely on, this may be even more important for online government resources. For public health professionals in government, they may also wish to consider how they approach engaging with demographics who have lower levels of education, as they appear less reliant on government sources of information and may respond better to other non-government sources of influence.

If women are expected to engage in the cognitive labor of managing COVID-19 information sources, this adds further demands to their lives. Unequitable cognitive labor compounds existing disparities in care work (27–30) and the complications of COVID-19. This overburdening may be detrimental to the emotional, physical, and cognitive wellbeing of women, and may negatively mediate their experience of public health information seeking, such as through seeking quick and easy sources, leaving them vulnerable to misinformation. Given that women also act as gatekeepers for health information (31–33), it is important that cognitive labor is further investigated and its impacts gauged and appropriately responded to by public health practitioners. Aiming to alleviate existing structural disparities, designing messaging that accounts for cognitive labor and women's contexts, and finding ways to identify excessive or unnecessary cognitive labor in frontline engagements, are all potential practical pathways if increased cognitive labor is identified. An alternative solution would be to empower alternative members of social groupings to become gatekeepers for this information through improving engagement and self-efficacy of these groups.

Our study does not claim to be representative of all women, but instead signposts experiences potentially important in understanding public health information seeking in context, but which requires further investigation to validate. While we note the potential implications of cognitive labor, and call for greater investigation into its effects, we acknowledge limitations to our research. The qualitative sample was skewed, and whilst it did yield important insights into women's experiences, we have attempted to position the significance of these findings within this limitation. We further wish to emphasize that the low number of male participants means these results should be treated cautiously, and as indicative of a potentially important area for public health research and practice. This should not be considered as a generalizable truth on the experiences of COVID-19 information seeking behaviors, or the relationship of these with gender. Furthermore, given that female participants are more likely to complete survey research, there is the possibility of a cofounding variable at play, although this does not detract from the qualitative stories that participants shared. Further research is required to validate the gendered nature of cognitive labor, including studies that better capture male and gender-diverse populations. Despite these limitations, we positively associate the validity of this study with emerging research highlighting the gendered nature of labor in the context of COVID-19 (24, 25), and on the gendered nature of information seeking behaviors (34–36).

## Conclusion

Participants' listed government sources, followed by mainstream media, professional sources, and familial sources, as their preferred health information sources for COVID-19. Use of government sources varied depending on education level, with mainstream media use varying depending on employment status. The qualitative data collected and thematically analyzed here captured key themes around how people seek information relating to COVID-19. Our findings have generated a potentially novel direction of further investigation on cognitive labor, which may mediate public health information seeking. Further research on cognitive labor, healthcare, and COVID-19 will improve the rigor of this initial finding and develop practical actions that might be used to support both better health information seeking, and a more equitable relationship between gender, healthcare, and information seeking.

## Data availability statement

The datasets presented in this article are not readily available because sharing of qualitative datasets potentially violates ethics and privacy requirements for this research, as there is the potential for identifiable data to be shared. Sharing of quantitative datasets may be possible with checks to ensure privacy and ethical compliance. Requests to access the datasets should be directed to emily.brindal@csiro.au or ashlin.lee@csiro.au.

## Ethics statement

The study was assessed and approved by the CSIRO Human Research Ethics Low Risk Review Panel (#2021_105_LR). Written, informed consent was given by participants for this research. The patients/participants provided their written informed consent to participate in this study.

## Author contributions

AL: background research, conceptualization, qualitative data analysis and write up, and manuscript writing and rewriting. NK: manuscript writing and rewriting, quantitative data analysis and write up, critical feedback, and conceptual development. LH: critical feedback, editing, and proofing. AR: critical feedback. EB: critical feedback, quantitative data analysis, and table building. All authors contributed to the article and approved the submitted version.

## Footnotes

^1^Note that coding frequency refers to the number of unique references made in a participant's statement. Depending on the length and depth of statements, multiple references may be appropriate. This means that it is possible for the number of coding references to exceed the number of participants, as is the case with Question 3. The purpose of referring to this is to indicate that female participants not only provide more text, but that their responses were of greater qualitative depth, raising more themes than male participants.
