# Supplementary material for: The invisible burden of managing COVID-19 for Australian women: Cognitive labor and public health information
Source: Front Public Health. 2023 Feb 1;11:1041944. doi: 10.3389/fpubh.2023.1041944 (PMC9929454; doi:10.3389/fpubh.2023.1041944)
Supplement: Supplementary file 1 [file Table_1.DOCX]

Supplementary Material

The invisible burden of managing COVID-19 for Australian women: Cognitive labour and public health information

Ashlin Lee ^1,2,3^, Naomi Kakoschke ^4^, Liesel Higgins ^5^, Andrew Reeson ^6^, Emily Brindal ^4^

^1^Environmental Informatics Group, Land and Water, Commonwealth Science and Industrial Research Organisation (CSIRO), Canberra, Australian Capital Territory, Australia

^2^ Justice and Technoscience Lab (JusTech), School of Regulation and Global Governance, The Australian National University, Canberra, Australian Capital Territory, Australia

^4^ Human Health, Health & Biosecurity, Commonwealth Science and Industrial Research Organisation (CSIRO), Adelaide, South Australia, Australia

^5^Australian e-Health Research Centre, Health & Biosecurity, Commonwealth Science and Industrial Research Organisation (CSIRO), Brisbane, Queensland, Australia

^6^ Humans and Machines, Data61, Commonwealth Science and Industrial Research Organisation (CSIRO), Canberra, Australian Capital Territory, Australia

*** Correspondence:**Ashlin Lee
Ashlin.lee@csiro.au

# Supplementary Data – Questionnaire

| - **Questions** | **Response Option** |
| --- | --- |
| **Demographics (displayed once only)** | |
| 1. What sex do you identify with? | Male/Female/Non-binary/Prefer not to answer |
| 1. What is your current age in years? | 18-24  25-29  30-34  35-39  40-44  45-49  50-54  55-59  60-64  65-69  70-74  75-79  80-84  85+ |
| 1. Were you born in Australia? | Yes/no |
| 1. How would you describe your marital status? | Single; defacto/married; divorced; widowed |
| 1. Do you have children living with you?* | 1. Yes/no 2. If yes to question 5a: Enter number 3. Are these children currently at school? Yes/no/some |
| 1. What postcode do you live at? | Enter number |
| 1. Highest level of education completed (from ABS) | Year 9 or below  Year 10  Year 11  Year 12  Certificate 2 or 3  Certificate 4 or 5  Diploma or Advanced Diploma  Bachelor/Undergraduate Degree  Graduate Diploma/Graduate Certificate  Postgraduate Degree  Certificate not further defined  Level not determined |
| 1. Employment status | Part-time, full-time, casual, unemployed |
| * Note: only survey respondents who answer ‘Yes’ to question 5a will be shown content relevant to families with children | |
| **Evaluation questions repeated for each content section** | |
| 1. Did you read the content presented? | yes/no/some of it |
| 1. How well did you understand the content? | scale 1-10; 1 = not at all, 10 = extremely well |
| 1. How relevant was the content to you? | scale 1-10; 1 = not at all, 10 = extremely relevant |
| 1. How likely are you to use this information in the future? | scale 1-10; 1 = not at all likely, 10 = extremely likely |
| **General questions** | |
| 1. What do you think is the best option for presenting this information? | Checkbox options:   - Website; - Printed brochure; - Smartphone application; - Other (enter) |
| 1. How likely would you be to recommend this information to a friend? | scale: 1-10; 1 = not at all likely, 10 = extremely likely |
| 1. Is there anything else you would like to provide feedback on? For example, consider the images used, the language style and the structure | Open-ended question |
| 1. What other sources of information do you use to learn about COVID-19? | Please select as many as relevant from the following:   - - Australian government sources   - Non-Australian government sources   - Mainstream media (ABC, SBS, Channel 7, Channel 9 etc)   - Other media sources (please specify)   - Professional sources (medical authorities, professional bodies etc)   - Online sources (Please specify)   - Community sources (community leaders, religious figures, etc) (please describe)   - Personal or familiar sources (friends, family etc)   - Celebrities, personalities, or specific figures (please describe)   - Other (please specify)   - None |
| 1. What is your preferred method of accessing information from these sources? | Please select as many as relevant from the following:   - - Face to face (talking with family, friends, community leaders etc)   - Radio - Podcast   - Television   - Printed media (newspaper, magazines etc)   - Webpages/ online   - Search engines (eg Google)   - Social media platform (Youtube, Facebook, Twitter etc)   - Social, mobile, or digital messaging service (WhatsApp, Instagram, TikTok, Discord etc)   - Other (please specify) |
| 1. Why do you use these sources? | Open-ended question |
| 1. How have you used these sources to prepare for COVID-19 related events? | Open-ended question |
| 1. Have you changed the way you seek out information in response to COVID-19 since the start of the pandemic? | Yes/no  If yes, please describe how/why |
| 1. Would you be willing to be contacted for a brief follow up (i.e., 5–10-minute) phone conversation? | Yes/no  If Yes: Please email us with your name and best contact method at [**motivation@csiro.au**](mailto:motivation@csiro.au) |
